# Intraoperative hypotension is a risk factor for postoperative acute kidney injury after femoral neck fracture surgery: a retrospective study

**DOI:** 10.1186/s12891-019-2496-1

**Published:** 2019-03-27

**Authors:** Woo Young Jang, Jae-Kyun Jung, Dong Ki Lee, Seung-Beom Han

**Affiliations:** 1 Department of Orthopaedic Surgery, Anam Hospital, Korea University College of Medicine, 73 Inchon-Ro (Anam-dong 5-ga), Seongbuk-gu, Seoul, 02841 South Korea; 2 Department of Orthopaedic Surgery, Suyu Danaeun Jeonghyeongoegwa , Dobong-ro 320, Seoul, Gangbuk-gu 01062 South Korea

**Keywords:** Acute kidney injury, Femoral neck fractures, Intraoperative hypotension

## Abstract

**Background:**

Hip fracture in elderly patients is a serious health concern due to the associated morbidity and mortality. Although acute kidney injury after hip fracture is known to be a significantly poor prognostic factor for morbidity and mortality, the literature regarding the risk factors for acute kidney injury after hip fracture is insufficient. This study aimed to investigate the incidence and associated risk factors for acute kidney injury in patients with femoral neck fracture.

**Methods:**

A total of 248 patients who underwent an operation for femoral neck fracture between January 2011 and January 2015 were retrospectively analyzed. Acute kidney injury was defined according to the Kidney Disease: Improving Global Outcomes guidelines.

**Results:**

The incidence of acute kidney injury was 17.7% (*n* = 44). Risk factors for acute kidney injury included diabetes mellitus, pre-existing renal disease, preoperative blood urea nitrogen (BUN), preoperative estimated glomerular filtration rate (eGFR), preoperative haemoglobin (Hb) level, type of operation, postoperative creatinine level and intraoperative hypotension (*P* <  0.05). After controlling for confounding variables, intraoperative hypotension was only the independent risk factor for acute kidney injury (*P* = 0.012).

**Conclusions:**

Acute kidney injury was found to occur frequently after surgery for femur neck fracture. Surgeons should be aware of acute kidney injury when planning the management of patients with femoral neck fracture and consider that the duration of intraoperative hypotension is a risk factor for acute kidney injury.

## Background

Hip fracture in elderly patients is a serious health concern due to the associated morbidity and mortality [1–4]. Complications such as deep vein thrombosis, pulmonary embolism, and myocardial infarction after hip fracture increase morbidity and mortality [5]. Of these, acute kidney injury (AKI) after hip fracture is known to be a significantly poor prognostic factor for morbidity and mortality. AKI is characterized by the sudden impairment of kidney function and can lead to potentially catastrophic complications after hip fracture surgery [2, 6, 7]. Although the exact definition of AKI has been subject to debate, a consensus definition of AKI according to the Kidney Disease: Improving Global Outcomes (KDIGO) guidelines was established in 2012 and is widely used [8].

While several studies have reported that the postoperative development of AKI is associated with high morbidity and mortality after orthopedic operation, data regarding the risk factors for AKI after hip fracture are insufficient. Furthermore, the definition of AKI applied in the existing studies did not correspond to that specified by the KDIGO guidelines. Therefore, the purpose of this study was to investigate the incidence of and associated risk factors for AKI according to KDIGO guidelines in patients with femoral neck fracture.

## Methods

This study was approved by our institutional review board. We retrospectively reviewed 312 patients who underwent surgery for femoral neck fracture between January 2011 and January 2015. All fractures were operated on by a single expert orthopedic surgeon. Of these, 24 patients were excluded due to insufficient medical records, and 36 patients were excluded as they were less than 65 years old. Finally, 248 patients (66 men and 182 women) were enrolled in this study. The mean age of the patients at operation was 77.6 years (range, 65–97 years) and the patients were followed for an average of 21.32 days (range, 5–96 days).

Demographic and clinical data including past medical history were thoroughly reviewed. The American Society of Anesthesiologists (ASA) score was determined to evaluate the general comorbidity of patients. Use of medications, such as angiotensin-converting enzyme inhibitors (ACE-Is), non-steroidal anti-inflammatory drugs, and contrast nephrotoxic antibiotics, which are known to affect renal function, was recorded, and preoperative hemoglobin (Hb) levels, electrolyte levels including blood urea nitrogen (BUN), and serum creatinine (SCr) were measured.

Additionally, intraoperative risk factors for AKI were assessed on the basis of the type of operation, operation time, intraoperative blood loss, and the presence of intraoperative hypotension (systolic blood pressure < 80 mmHg or a mean blood pressure < 55–60 mmHg) that persisted for more than 5 min [9].

AKI was diagnosed if any one of the following conditions was present, according to KDIGO 2012 [8]: an increase in SCr of ≥0.3 mg/dL (≥ 26.5 μmol/L) within 48 h, an increase in SCr of ≥1.5 times the baseline value within 7 days, or urine volume <  0.5 mL/kg/h for 6 h. SCr levels were measured at baseline and periodically over 3 days following the operation.

### Statistical analysis

Patients were subdivided into 2 groups according to the presence or absence of AKI: the AKI and non-AKI groups. Quantitative variables are expressed as means ± standard deviation (SD) and qualitative variables as number and percentage. Student’s t-test was used for the comparison of quantitative variables and Fisher’s exact test was used for qualitative variables. We performed multivariable logistic regression analysis using the significant variables revealed by the univariate analysis as the independent variables and the presence of AKI as the dependent variable. The stepwise backward elimination method was used for multivariable logistic regression and the final model was selected according to the Akaike’s information criterion (AIC) value. All statistical analyses were conducted using SPSS software (IBM SPSS, version 21; SPSS, Chicago, IL) and a *P*-value < 0.05 was considered statistically significant.

## Results

The demographic and clinical characteristics of patients are presented in Table 1. The overall incidence of AKI was 17.7% (*n* = 44) and the AKI group had a significantly higher rate of medical comorbidities, including diabetes (*P* <  0.001), pre-existing renal disease (*P* <  0.001), and medication with an ACE inhibitor (*P* = 0.037) than the non-AKI group. Preoperatively, the AKI group had a significantly lower preoperative Hb (*P* <  0.001), BUN (*P* <  0.001), preoperative (*P* = 0.034) and postoperative creatinine (*P* <  0.001), eGFR (*P* <  0.001), proteinuria (*P* = 0.029), ESR (*P* <  0.001), and CRP (*P* = 0.012) levels.

**Table 1:** Baseline characteristics of patients included in the study

Variable	AKI (*n* = 44)	Non-AKI (*n* = 204)	*P*-value
Demographic characteristics			
Gender (male/female) (n, %)	12 (27.3)/32 (72.7)	54 (26.5)/150 (73.5)	0.915
Age (years)^a^	79.0 ± 7.8	77.3 ± 6.9	0.153
Length of hospital stay (days)^a^	27.6 ± 6.8	19.7 ± 9.0	0.002^*^
Type of operation (n, %)			0.015^*^
Bipolar hemiarthroplasty	39 (83.0)	158 (66.7)	
THR	7 (14.9)	41 (17.3)	
Osteosynthesis	1 (2.1)	38 (16.0)	
Comorbidities (n, %)			
Hypertension	25 (56.8)	106 (51.9)	0.560
Diabetes mellitus	23 (52.2)	50 (24.5)	< 0.001^*^
Previous renal disease	15 (34.1)	25 (12.3)	< 0.001^*^
Previous heart disease	8 (18.2)	35 (17.2)	0.775
Medication history (n, %)			
ACE inhibitor	22 (50.0)	68 (33.3)	0.037^*^
NSAID	5 (11.4)	14 (6.9)	0.311
Contrast	2 (4.6)	26 (12.7)	0.120
Nephrotoxic antibiotics	2 (4.6)	2 (1.0)	0.089

*Abbreviations*: *ACE* angiotensin-converting enzyme, *AKI* acute kidney injury, *NSAID* non-steroidal anti-inflammatory drug, *THR* total hip replacement

^a^Values are presented as mean ± standard deviation

^*^Statistically significant between the 2 groups (*P* <  0.05)

**Fig. 1:**
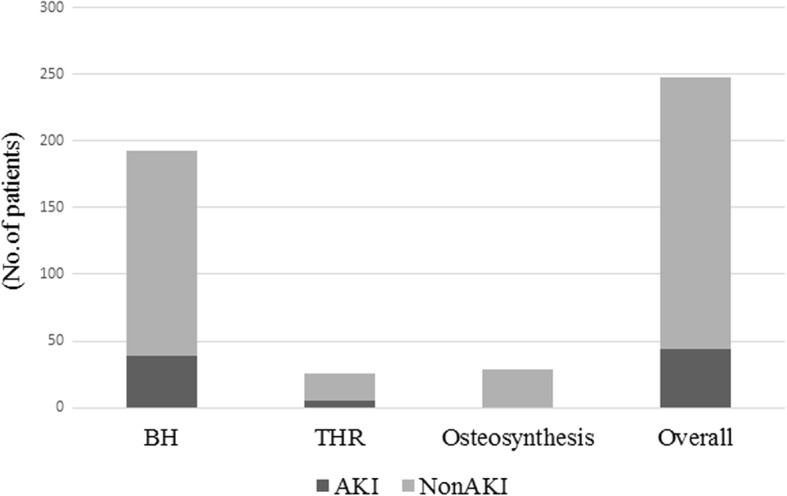
Prevalence of AKI in different types of operation. The incidence of AKI was significantly higher in patients who underwent bipolar hemiarthroplasty than in those who underwent other types of operations

The type of operation differed significantly between the AKI and non-AKI groups (*P* = 0.028). In a subgroup analysis according to the type of operation, including bipolar hemiarthroplasty (BH), total hip replacement (THR), and osteosynthesis, the prevalence of AKI was significantly different (Fig. 1). The incidence of intraoperative hypotension was significantly higher in the AKI group than in the non-AKI group (*P* = 0.42, Table 2).

**Table 2:** Baseline laboratory and hemodynamic variables in the AKI and non-AKI groups

Variable	AKI (*n* = 44)	Non-AKI (*n* = 204)	P-value
Hemoglobin (g/dL)^a^	11.1 ± 1.5	12.1 ± 1.4	< 0.001^*^
Sodium (mg/dL)^a^	136.6 ± 4.0	137.4 ± 9.7	0.558
Potassium (mg/dL)^a^	4.0 ± 0.5	4.1 ± 0.4	0.521
BUN (mg/dL)^a^	26.4 ± 18.5	18.5 ± 8.7	< 0.001^*^
Preoperative serum Cr (mg/dL)^a^	1.4 ± 1.0	1.0 ± 1.0	0.034^*^
Postoperative serum Cr (mg/dL)^a^	2.4 ± 1.0	1.0 ± 0.8	< 0.001^*^
eGFR (mL/min per 1.73 m^2^)^a^	60.7 ± 32.3	77.0 ± 26.1	< 0.001^*^
Proteinuria (n, %)	14 (31.8)	33 (16.2)	0.029^*^
ESR (mm/h)^a^	30.0 ± 20.4	18.9 ± 15.2	< 0.001^*^
CRP (mg/dL)^a^	39.3 ± 49.5	23.2 ± 34.9	0.012^*^
Time of operation (min)^a^	55.0 ± 15.4	60.2 ± 20.1	0.104
Intraoperative blood loss (mL)^a^	523.1 ± 226.9	485.3 ± 153.4	0.472
ASA scores			0.203
II	109 (53.4%)	17 (38.6%)	
III	91 (44.6%)	26 (59.1%)	
IV	4 (2.0%)	1 (2.3%)	
Intraoperative hypotension (n, %)	26 (59.1)	91 (44.6)	0.042^*^
Postoperative blood loss (mL)	260.5 ± 270.5	246.9 ± 184.6	0.707

*ASA* American Society of Anesthesiologists, *BUN* blood urea nitrogen, *Cr* creatinine, *CRP* C-reactive protein, *eGFR* estimated glomerular filtration rate, *ESR* erythrocyte sedimentation rate

^a^Values are presented as mean ± standard deviation

^*^Statistically significant between the 2 groups (*P* < 0.05)

Univariable logistic regression analysis showed that type of operation, diabetes mellitus, pre-existing renal disease, ACE inhibitor, preoperative Hb level, preoperative BUN level, preoperative eGFR, postoperative Cr level and intraoperative hypotension were the significant risk factors for postoperative AKI. According to multivariable logistic regression analysis controlling for confounding variables, the presence of intraoperative hypotension was the only significant risk factor for postoperative AKI (Table 3).

**Table 3:** Multivariable logistic regression analysis for acute kidney injury

Multivariable analysis			
Variables	OR	95% CI for OR	*P*-value
Type of operation	0.33	0.09–0.94	0.064
Diabetes mellitus	2.36	0.80–7.01	0.177
Previous renal disease	2.57	0.60–3.24	0.598
ACE inhibitor	1.43	0.50–1.17	0.857
Hemoglobin (g/dL)	1.43	0.50–1.17	0.214
BUN (mg/dL)	1.03	0.99–1.08	0.053
eGFR (mL/min per 1.73 m^2^)	1.02	0.99–1.04	0.181
Intraoperative hypotension (n, %)	5.14	1.54–20.35	0.012^*^

*Abbreviations*: *CI* confidence interval, *OR* odds ratio, *ACE* angiotensin-converting enzyme, *BUN* blood urea nitrogen, *eGFR* estimated glomerular filtration rate

^*^Statistically significant between the two groups (*P* < 0.05*)*

## Discussion

AKI develops in 7.5% of patients undergoing noncardiac surgery [10] and the postoperative development of AKI is associated with an eight-fold increased probability of death within 30 days of surgery [11]. Interestingly, the previously reported incidence of AKI in patients undergoing cardiac surgery varies between 4 and 9% [12, 13], compared to between 15.3 and 16% for hip fracture surgery [2, 7]. Despite the significant association between AKI and hip fractures, the importance of AKI in patients with femoral neck fracture has only recently received attention. In the present study, the incidence of AKI in patients who underwent femoral neck fracture surgery was 17.7%, which was consistent with previous reports [2, 7]. There was a significant difference in the prevalence of diabetes, pre-existing renal disease, preoperative Hb level, preoperative BUN level, preoperative Cr level, type of operation, and intraoperative hypotension between the AKI and non-AKI groups. After controlling for confounding variables, intraoperative hypotension was the only independent risk factor for AKI.

Bipolar hemiarthroplasty, rather than THR, was significantly associated with an increased risk of AKI in this study, although THR leads to massive blood loss and prolonged operating time because of its invasive and meticulous surgical technique. One possible explanation is that the patients who underwent bipolar hemiarthroplasty were typically older and less ambulatory, owing to the presence of underlying disease. Several studies have demonstrated that the incidence of AKI increases with age and is most common in elderly patients [14, 15]. Additionally, diabetes and pre-existing renal disease have emerged as independent predictors of AKI [16]. Our study also showed independent risk factors, such as laboratory findings (BUN, postoperative serum creatinine, ESR) and diabetes, associated with AKI after femoral neck fracture surgery. Therefore, surgeons should be aware of the possibility of AKI when treating femoral neck fracture in patients with high morbidity.

The use of hypotensive anesthesia is generally recommended in the orthopedic department for decreasing intraoperative blood loss, transfusion requirements, and operation time, improving the quality of the surgical field, and preventing deep vein thrombosis. However, it may lead to intraoperative dehydration and hypotension and dehydration may in turn cause endothelial injury and the subsequent local release of endothelin, angiotensin II, and catecholamines, all of which cause vasoconstriction and exacerbate ischemia in the kidney [17, 18]. A clear relationship between hypotension during operation and the development of AKI was observed in this study. Therefore, clinicians should consider intraoperative hypotension among the various risk factors for AKI and prioritize intraoperative blood pressure control.

The major limitation of this study was that it did not investigate the total duration of intraoperative hypotension. Although the duration of hypotension required to incur damage is unclear, interest in the effects of the duration of intraoperative hypotension on AKI has recently increased [9, 19]. In addition, there are almost 50 different definitions of intraoperative hypotension in the recent literature [20], which may reduce the generalizability of our findings, although we used the most common definition [9]. Thus, future investigations that establish a clear definition of intraoperative hypotension and determine the critical duration of intraoperative hypotension are warranted.

## Conclusions

In conclusion, AKI was found to occur frequently after surgery for femur neck fracture. Surgeons should be aware of acute kidney injury when planning the management of patients with femoral neck fracture and consider that the duration of intraoperative hypotension is a risk factor for acute kidney injury.

## Abbreviations

ACE-Is: Angiotensin-converting enzyme inhibitors
AKI: Acute kidney injury
ASA: American Society of Anesthesiologists
BH: Bipolar hemiarthroplasty
BUN: Blood urea nitrogen
Hb: Hemoglobin
KDIGO: Kidney Disease: Improving Global Outcomes
SCr: Serum creatinine
THR: Total hip replacement
